# Indents

**Published:** 1920-06

**Authors:** 


### INDENTS

**☞Brief Articles of Technical or Scientific Value will receive notice and comment. Contributions invited.**

---

English Registered Dentists. The English Dental Register records for 1920 5,455 names, being 112 less than last year, and only 315 more than seven years ago. From 1916 to 1918 there were barely 150 dental students matriculated. The diploma of L. D. S., England, is held only by 2,498 registered dentists. 657 possess the Edinburg diploma; 528, Glasgow; 460, Ireland; 92, Manchester; 63, Liverpool; 15, Leeds; 13, Durham; 12, Birmingham. Eighty per cent of those on the Register are licentiates or graduates in Dental Surgery.

---

Setting Plaster. Dr. Dayton D. Campbell claims that plaster sets harder and quicker if potassium sulphate is added to the plaster when mixing. This may be true in some cases. Plaster will not set as quickly without sulphate, but it should not be used excessively, or dried out too soon after pouring. Salt is not so expensive and will serve all practical purposes for impressions, if the impression mass is not used in great bulk. In thin layers plaster sets hard enough for ordinary impressions and will not change form. Plaster will secure a good surface. It should not be used for casts needing strength.

---

The Short and Catchy Proprietary Name. A laborer went to a Brooklyn physician for treatment and was given three prescriptions. One of the prescriptions, according to the *Food and Drug Bulletin* of the Department of Health, City of New York, called for ''Laxol,'' the word being written on a piece of blank paper without directions. The drug clerk misread the prescription and dispensed an ''original'' bottle of ''Lysol'' which bore the usual poison label with skull and crossbones. The man drank the entire three ounces of Lysol and died half an hour later. The case is now in the hands of the District Attorney, the drug clerk being held under $10,000 bail. "Laxol," as our readers know, is castor oil sweetened with saccharin and flavored with peppermint. There is no excuse for prescribing the product. The official Aromatic Castor Oil (Ol. Ricin. Arom.) of the National Formulary would answer every purpose served by the proprietary preparation.—*Journal A. U. A.*, May, 1920.

---

A Recipe for Old Age. In the memoirs of Baron de Grimm, written between the years 1753 and 1790, appears an account of the death of a member of the French Academy, a physician of eminence, one John J. de Mairen, at the age of 93. "He had arrived at this great age," say the memoirs, "without any infirmity, and preserved his good looks, his activity, as well as the entire use of his faculties to the last moment of his life." Until his last illness he scarcely missed going out a single day; he lived in the best society, dined out almost every day, and passed the afternoon in making visits and the evening among his books. "He was exactly the kind of a person to live to a great age," says de Grimm. "His head was well formed, he had great equanimity of temper, great moderation in his passions, or rather he was destitute of passions; he had sensibility enough to engage the regard of those with whom he associated, and to contract those ties of intimacy which were sufficient for him, which have not indeed the charms of friendship, but which do not draw after them the same obligations. He had not warmth of heart enough to feel the necessity of an attachment which rules despotically; of a friend who disposes of us at pleasure, who forms the happiness or the misery of our lives. He had much prudence and foresight, paid great attention to himself, and was very methodical in whatever he did." Is this a text on geriatrics?—*Journal A. U. A.*, May, 1920.

Obtunding Dentine. A combination of chloroform, ether, and menthol applied with a hot air syringe makes a reliable application for controlling sensitive dentine. Don't apply the remedy too hot or with a strong blast of the heated drug.—*Oral Health.*

---

Discussing Papers. There are two men in the profession, one a specialist, the other a critic, who were always crossing swords whenever the opportunity offered. Their remarks were usually aimed at each other rather than the subject under discussion. It tended to hurt more than help both of them in the minds of other men. The specialist was invited to read a paper before a small society of the younger men, and the critic had been invited there to discuss it. The young men evidently expected the usual fireworks. It so happened, however, that the two men chanced to meet and made the journey to the appointed place together. Being good fellows and friendly in private, they talked the matter over and admitted that they did agree upon all of the essentials of the subject to be presented, and decided that instead of having their usual quarrel over trifles, they would support each other as far as possible, in the hope of giving these younger men a clearer understanding of the subject. The result was that the young men were unanimous in declaring it the most instructive meeting they had ever attended.

What an improvement it would be if this idea could be followed at all dental meetings!—Dr. F. C. Brush, in May *Cosmos.*

---

Curriculum for Hygiene Schools. I know that in each city and each center where the schools form, those in charge will have their own ideas of what this field of service shall be, and it will be very difficult to change their ideas; but at the beginning of this course for the dental hygienist it was our conception that she should be a worker for the prevention of disease. The term originally was "the dental nurse," but in my analysis of the field of service or endeavor in which we wanted to launch these women to do so much for humanity, I felt the one thing she was not to do was to treat disease, but to fight against disease. She was to be a means for the prevention of dental caries, and a teacher of hygiene. In my announcement of 1913 that was placed in the notice—that it was to be our endeavor to teach these women to be hygienists, and they were to be known as dental hygienists.

I agree with Dr. Kirk that you can not have too extensive a course, or know too much. The salvation of the world is hinged on one word—education. The more you know, the more you will keep out of trouble, and that is the one thing the corps in the public schools are doing—endeavoring to prevent physical defects by teaching hygiene.

It is my hope that the courses as they are being established now throughout the country, shall be planned with the view that these women shall be known as general hygienists, and that mouth hygienists make for the most important part of general health—in the prevention of disease.—Dr. Fones, in *Cormos.*

---

THE RELATION OF TEETH TO OCULAR DISORDERS. I am not infrequently asked to give an opinion on this subject, and hope dental surgeons will give expression of their experiences. I do not now refer to the effect of such teeth in causing, for example, facial neuralgia or certain reflex symptoms, but whether they can originate an inflammatory disease of the eye, for instance, a uveitis. In my own experience I have never observed a uveitis, for example, which could be traced to a non-infected, impacted molar, although I have once quite recently studied a patient with one of the forms of unilateral retrobulbar optic neuritis with such a molar on the same side, where the ocular disorder rapidly disappeared after removal of the tooth. It is, however, fair to state that much prior treatment had started the patient well on the way to recovery, which might therefore very well have taken place without the elimination of the buried tooth. Evidently the condition of the impacted tooth and its surroundings must largely determine the need of surgical interference.

My reason for referring especially to this part of the subject is that there seems to be a good deal of difference of opinion as to the harmful effects of impacted teeth, on the one hand, and on the other of the tendency to ascribe to them influences which they do not possess. Thus, I have heard a prominent dental surgeon urge the removal of an impacted molar for the relief of choked disk due, as it was proved by autopsy and evident by the clinical symptoms, to a tumor of the cerebellum. Recently I have examined two patients, one with a well-known type of choroiditis due to an equally well-known cause, and the other an old man with a typical so-called senile macular degeneration, where in each instance a buried tooth was insistently brought forward as the causal factor.

Whenever striking, not to say startling, advantageous results follow certain therapeutic procedures, there is a tendency on the part of those concerned to narrow their points of view, "to ride a hobby," as we are wont to express it. There are sure to develop, on the other hand, antagonisms, and neither those who recklessly advocate, nor those who stubbornly oppose, are correct. Each misses the path of safety and the value of scientific investigation followed by recommendations tempered with sound judgment.

There is no manner of doubt that many teeth have been needlessly sacrificed, and this is equally true of many appendices, many foreskins, many tonsils—the list is a long one. But he is just as wrong who disdainfully disregards careful dental examination because, forsooth, he chooses to regard the whole matter as a "fad," as is he who suspects every tooth and extracts too many. No sane physician, surgeon or dentist will fail to agree that the not infrequent unnecessary sacrifice of vital teeth should be condemned. As L. F. Barker has well said, "A tooth should not be sacrificed unless indications are clear for its removal, and widespread orders to extract teeth unless they are so diseased that they should come out should not be given." On the other hand, as he further says, to try and save teeth which can not be made aseptic is unwise, not to say foolish, for they may be, and the danger is a real one and has now many times been definitely demonstrated, the cause of general and local disease such as we have discussed this evening.

A few weeks ago I examined a comparatively young man who was literally toothless because he had a form of axial atrophy of the optic nerves, which was due to the abuse of alcohol and tobacco, and which yielded promptly to the usual measures—abstinence and elimination treatment. Again I studied a patient within the last year with a central scotoma apparently also due to a chronic retrobulbar neuritis, whose teeth were a marvel of the gold-filling art and of bridgework. It seemed almost a sacrilege to suspect them, but X-ray examination revealed what was beneath, and with the eradication of the covered-in septic areas the visual difficulties disappeared. It is not for me to dwell on the lesson which such a case teaches. I know how carefully this is being considered in scientific dental circles.

Speaking purely as an ophthalmologist I desire to say this, and I know my ophthalmic *confreres* will agree with me, namely, that the proper management of cases of uveal tract disorders, in particular of some forms of keratitis and of certain disorders of the retina and of the optic nerve, demands the co-operation of the physician, the ophthalmologist, the otolaryngologist and the dental surgeon. These cases must be studied from all standpoints, and sources of infection eradicated or sterilized. Co-operation is the master word in this business. We must act not along the lines of ill-considered and reckless sacrifice, but with due care and judgment, weighing the evidence as it presents itself from the investigation of all sources from which the etiological factor may spring and not from one alone. Working thus "in perfect sympathy and uncontending equity," we who are banded together for the relief of human suffering and the prolongation of human life shall achieve what must be our professional aim—the cure of those who seek our advice.—Dr. Schweinitz, in *Cosmos*, for May.

---

Government Grant to Royal College of Dental Surgeons. The Ontario Government has decided to give financial aid to the building programme of the Royal College of Dental Surgeons and $100,000 appears in the supplementary estimates for this purpose. The College, in enlarging its teaching facilities, has been largely actuated by a desire to meet the requirements of returned soldiers. Of 320 first-year students, 260 are returned men. In a total registration of 780, there are over 500 enlisted men, of whom 100 suffered disability. The Honorable R. H. Grant, Minister of Education, issued the following statement to the press:

"The new building and equipment cost $150,000, of which the college itself has paid $50,000. The work was not begun until after the whole problem had been laid before the Honorable Dr. Cody, and the building extension was undertaken with the full knowledge of the late Government. Had not the plans been proceeded with, the college would have been unable to receive one new student this year, the entire accommodation of the old building being barely sufficient for the students who had enlisted during their course and who were returning to complete their course. The seriousness of this situation may be appreciated when it is remembered that this institution is the only dental college in Ontario. This is the first request for funds ever made by this college, and it was for the Department of Education to determine whether assistance to dental education should be given direct to the college or through the universities. The Government has decided that it would be unwise to have a duplication of dental faculties in the Province, and that dental education will be served best by maintaining the present position of the Royal College of Dental Surgeons, which is now recognized as one of the foremost dental institutions in the world. Amendments to the Dentistry Act will be proposed at the present session, which will provide that the property of the College can not be disposed of without the consent of the Lieutenant Governor-in-Council, and that the Minister of Education for the time being will be ex-officio a member of the board of directors.''

The Royal College and the dental profession are to be congratulated upon this very generous and whole-hearted public recognition of the growing importance of dentistry and of its vital relationship to the health of the people.

The graduates of the College must feel a great sense of pride that their effort in establishing and administering a dental college, dating back over the past fifty years (without expense to the public throughout that whole period), should be officially recognized as a great public service.

The sympathetic and helpful attitude of the Honorable the Minister of Education of the Province of Ontario, of the Drury Cabinet and the members of the Legislature who supported this grant, is greatly appreciated by the entire dental profession of the Province.

The amendments referred to by the Honorable the Minister of Education, while giving public recognition to the College, will not in any way disturb the vested rights of the dental profession nor will they remove from the profession its present responsibility for the proper administration of the College through the Board of Directors elected by the Licentiates of the Province.—*Oral Health.*

For Erosion. A paste of milk of magnesia and prepared chalk, applied around the necks of the teeth before retiring, will remain longer than any preparation yet tried. Spread on a piece of thin muslin and placed under the lip at night, will be found efficient in very wet mouths.—*Oral Health.*

---

### My Dental Chair

I love it, I love it, and who shall dare
To chide me for loving my dental chair?
Long years has it stood in the self-same place,
Secure and firm on its oil-filled base;
'Tis viewed by many with anxious eye,
And its occupants change as the hours pass by,
For forms that are plump and forms that are spare
Have pressed the plush of my dental chair.

How well I remember my joyful pride
When first I stood at my new chair side,
For every patient its works I'd try,
I'd let it down and I'd pump it high;
I did my utmost to keep it neat,
I polished its levers and brushed its seat,
I oiled its joints with persistent care,
I was so proud of my dental chair.

Now and again, when its yielding seat
Supports the form of a maid petite,
I am sometimes nearly inclined to think
That the screws in its head-rest almost wink,
And it seems to ask with a knowing smile,
"How would my job suit *you* for a while,
With *your* arms entwining this form so fair?"
"Tut, tut, be quiet, you old dental chair."

At other times its ungrudged support
Is lent to forms of a different sort,
A bearded gaffer with senile fuss
May wish to be made edentulous;
And perhaps at the first submucous prod
He jumps from his seat and exclaims, "Good ———!"
I seize the old cove by his scanty hair:
"Sit still, you old fool, in my dental chair."

We are getting older, my chair and I,
As the patients go and the years pass by;
Our joints both creak as they stiff become,
While its seat is as bald as my cranium.
Quite soon I will come to the end of my toil,
When I shuffle off this poor mortal coil,
Dismiss my last patient with fatherly air,
And sit down and die in my dental chair.

Walter Rose, L.D.S., Sligo, in *Dental Record.*

---

The Middle of the Road. How easy it is in life to wander off into the hedges and ditches and to stray from the beaten path; what a difficult thing to stick to the middle of the road. We can not all travel a perfectly straight path owing to the diversities and distractions of life, but we can keep more nearly in line of our manifest duty than most of us do. It is human nature to constantly fly off at a tangent from the main purpose of life. We are not content to plod along on the straight and narrow way, but like cattle, when driven on the highway, we are forever exploring the fence corners and nosing in forbidden nooks. The novel, the strange, the unusual appeal to us, and divert us from the chief goal toward which all of our energies should be directed.

It seems so difficult for us to learn the lesson that in order to reach a given point it is always best to take the most direct route toward it, or at least the best beaten path. It is our love of diversion which distracts us and sends us into the by-ways. Some of this seems necessary and proper. In order to put zest into life we must have a certain amount of diversion. The dull routine of existence palls on the senses and makes us sluggish. It is said that variety is the spice of life, and yet too much spice is not good for anyone. Neither is too much diversion. If we season too highly our experiences with side issues we detract from the solid essentials of life, and get off the main beaten track.

If a man sets himself seriously to the accomplishment of a given purpose in life, and then keeps resolutely to the middle of the road leading to that purpose, there is hardly any set of circumstances that can prevent him from reaching his goal. But if this same man—wishing ever so fervently to accomplish his end—permits himself to be continually diverted by ulterior issues, he will never attain his purpose.

Constancy of aim is the keynote of achievement in most of the activities of life—moral as well as material. Mere goodness is not all, though goodness is one of the chief assets in shaping any career.

The first thing for every man to do is to make certain that he is on the right road, and when this is definitely determined, he should resolutely keep in the middle of that road and follow it to its logical terminus. In no way else shall he be assured of reaching his ultimate object.

I have watched many men and with much interest. I have seen some of them start out in the most brilliant way and with the utmost promise, only to fail lamentably in the end merely through being deflected from their chart by the magnetism of counter-attractions. It is so hard to be constant—it is so easy to be diverted. And I have seen other men who were not brilliant, men of less natural endowment, who have achieved success solely because they had constancy of purpose and the resolve to remain in the middle of the road.

And when one thinks of it, most roads are sufficiently winding and have ample change of scene for all legitimate entertainment if only we make the most of them as we pass along. No road is really dull. It is we who become dull, because we do not make note of all the wondrous phenomena laid out before our eyes. Thoreau's environment was narrow, or would have been narrow to most men, and yet what a world of interest he dug out of it. His country roads were never dull to him, no matter how many times he traveled the same way.

We can not all be Thoreaus, because we lack his vision, but we can all aim to get the most out of our particular niche in life by observing the beauties that are spread along the wayside of our existence, and we can see these beauties more surely and more safely if we remain in the middle of the road, and look at both sides as we journey on.—C. N. Johnson, in *Oral Health.*

